# Severe fever with thrombocytopenia syndrome virus infection during pregnancy in C57/BL6 mice causes fetal damage

**DOI:** 10.1371/journal.pntd.0008453

**Published:** 2020-07-30

**Authors:** Rui Chen, Zeng-qiang Kou, Xiao-rui Wang, Shu-han Li, Hai-lu Zhang, Zi-wei Liu, Dong Cheng, Zhi-yu Wang, Xue-Jie Yu, Hong-ling Wen

**Affiliations:** 1 Department of Microbiological Laboratory Technology, School of Public Health, Cheeloo College of Medicine, Shandong University, Key laboratory for the prevention and control of infectious diseases (key laboratory of China’s “13th Five-Year”, Shandong University), Jinan, Shandong Province, China; 2 Shandong Center for Disease Control and Prevention, Shandong Provincial Key Laboratory of Infectious Disease Prevention and Control, Jinan, Shandong Province, China; 3 Wuhan University School of Health Sciences, Wuhan, China; University of Liverpool, UNITED KINGDOM

## Abstract

**Background:**

Severe fever with thrombocytopenia syndrome virus (SFTSV) is a novel tick-borne phlebovirus, which is listed in the most dangerous pathogens by the World Health Organization, and has 12–30% fatality rates. SFTSV antibodies were reported in minks that experienced abortion or reproductive failure. The aim of this study was to determine whether SFTSV infection causes an adverse pregnancy outcome in the fetus using a pregnant mouse model.

**Methodology/Principal findings:**

We found SFTSV in the fetus after infection in pregnant mice, and some dams showed adverse pregnancy outcomes after infection with SFTSV including placental damage, fetal reabsorption, and fetal intrauterine growth restriction (IUGR). SFTSV had obvious tropism characteristics in the placenta, especially in the labyrinth. In early-gestation, pregnant mice infected with SFTSV had fetal IUGR and a high viral load in the fetus. The virus widely spread in infected fetuses, including the hindbrain, thymus, heart, spinal cord, and liver.

**Conclusions:**

Our study demonstrated that SFTSV was vertically transmitted to the fetus through the placental barrier of immunocompetent mice, and resulted in adverse pregnancy outcomes.

## Author summary

Severe fever with thrombocytopenia syndrome (SFTS) is an emerging infectious disease discovered in rural areas of China in 2009, caused by a novel tick-borne phlebovirus, the SFTS virus (SFTSV). At present, SFTS reported cases are increasing, and the epidemic area of SFTSV is expanding. SFTSV is currently endemic in China, Japan, South Korea and Vietnam, and has 12–30% fatality rates. This study demonstrated that SFTSV is vertically transmitted to the fetus through the placental barrier of immunocompetent mice, and can result in fetal damage. This study highlights the possible potential impact of SFTSV on pregnant women and livestock. The results suggested that we should actively monitor the pregnancy outcomes of women and livestock infected with SFTSV, and strengthen the study of the pathogenesis of fetal damage caused by SFTSV.

## Introduction

Severe fever with thrombocytopenia syndrome (SFTS) is an emerging viral hemorrhagic fever that is caused by a novel phlebovirus, the SFTS virus (SFTSV) [1]. SFTSV is currently endemic in China, Japan, South Korea, and Vietnam [2–4]. It is transmitted through tick bites and from person-to-person [5–9], and causes infection in thousands of people every year in China with annual case numbers increasing, and with a reported case fatality rate among hospitalized patients from 12% to 30% in China and 20% in South Korea and Japan [10–12]. SFTS mainly manifests as fever, thrombocytopenia, gastrointestinal symptoms, leukopenia, and elevated serum hepatic enzymes. Patients with severe illness usually die of multiorgan failure [1]. Many wild and domestic animal species such as dogs, goats, and cattle in SFTSV epidemic areas have a high seroprevalence of SFTSV antibodies [13, 14]. Animals infected with SFTSV usually have no obvious symptoms and viremia.

Rift Valley fever virus (RVFV), which belongs along with SFTSV to the genus *Phlebovirus*, was shown to be vertically transmitted and cause abortions and birth defects in ruminants (such as cattle, sheep, and camels) and humans [15, 16]. In addition, a recent report indicated that SFTSV antibodies were present in minks that had experienced abortion or reproductive failure [17]. However, it is not clear if SFTSV vertically transmits through the placental barrier and causes fetal damage. In this study, we used a mouse model to determine whether SFTSV can directly transmit through the placental barrier and cause fetal damage.

## Methods

### Ethics statement

The study was reviewed and approved by the ethics committees of Shandong University (Approved protocol no.20180313). All procedures involving animals in this study were in accordance with the guidelines of the National Institutes of Health.

### Viruses and cells

The SFTSV strain JS2011-013-1(GenBank: KC505126 to KC505128) was used in this study. JS2011-013-1 was isolated from human serum by the Jiangsu CDC and donated to our laboratory. The virus was propagated on Vero cells (Verda reno) in Dulbecco’s modified Eagle’s medium (DMEM) (Gibco, Grand Island, New York, USA) supplemented with 10% fetal bovine serum (FBS) (Gibco, Grand Island, New York, USA). The infectious dose of the SFTSV stock solutions was determined by calculating the 50% tissue culture infectious dose (TCID_50_) in Vero cells through the visualization of infection by an indirect immunofluorescent assay (IFA), as previously described [18]. Cells infected with SFTSV reacted with a human anti-SFTSV Gn protein monoclonal antibody (mAb)[19], followed by staining with fluorescein isothiocyanate (FITC)-conjugated goat anti- human IgG (H-L) (Proteintech, Chicago, Illinois, USA).

### Mouse infection experiments

C57BL/6 WT mice were purchased from Beijing Huafukang Bioscience. Eleven week-old mice were set up for timed mating. The pregnant mice were divided into 10 groups and intramuscularly inoculated with 100μl of SFTSV diluted in DMEM whereas the mock group of mice were inoculated with 100μl stroke-physiological saline solution (Table 1). Mice were inoculated in early-gestation [embryonic day 5 (E5)] and late-gestation (E14). Mouse weights and clinical signs were recorded every two days to eliminate non-pregnant mice.

**Table 1:** Grouping table of pregnant mice.

	Early-gestation	Late-gestation
	(E5)	(E14)
Mock	n = 5	n = 5
High dose (10^6^TCID_50_)	n = 5	n = 5
Low dose (10^3^TCID_50_)	n = 6	n = 6
Low dose^C^ (10^3^TCID_50_)	n = 5	n = 6
Mock^C^	n = 5	n = 5

^C^ were treated with Mitomycin C

Except for one dam per group that progressed to full-term and delivered pups on E19, the other groups were sacrificed on E17 [12 or 3 days post-infection (dpi)]. After delivery, dams and pups were sacrificed on day 1-post-delivery (15 or 6 dpi) to reduce consumption of newborn pups by the dam. After collecting blood through the orbital sinus, the maternal spleens, fetal placentas, and viscera (all viscera in the abdominal cavity, including the spleen, kidney, liver, and intestines) were immediately collected. In addition, the size of each intact fetus was measured at E17, as well as the crown-rump length (CRL) × occipito-frontal (OF) diameter of the head.

### Treatment with Mitomycin C

The immunosuppressive drug treatment on C57/BL6 pregnant mice was performed by intraperitoneal (IP) injection of mitomycin C daily, with 0.02 mg per mouse beginning 3 d before the infection and with 0.001 mg per mouse and continuing for 3 d after the infection [20].

### Detection of antibodies to SFTSV

The blood of pregnant mice was placed at 4°Cfor 16 h, then centrifuged at 4°C, 3000 g for 10 min, and the upper serum was collected. Anti-SFTSV total antibodies were detected by using a SFTSV ELISA Kit (Xinlianxin Biotech CO., LTD, Wuxi, China) according to the manufacturer’s instructions. Briefly, 96-well plates coated with recombinant SFTSV-proteins antigens and undiluted mouse serum were used for detection of anti-SFTSV total antibodies, with HRP-conjugated recombinant SFTSV-proteins antigens used to visualize the results. The optical density (OD) value of each ELISA was read using an enzyme-labeled instrument at 450 nm.

### Measurement of viral loads in the serum and tissues

Frozen tissues (maternal spleens, fetal placentas, and viscera of fetuses and pups) were homogenized with zirconia beads in TissueLyser II (Qiagen) in QIAzol Lysis Reagent (Qiagen). The tissue’s RNA was extracted using the RNeasy 96 QIAcube HT Kit (Qiagen) in QIAcube HT Instrument (Qiagen), according to the manufacturer’s instructions. The maternal serum RNA was extracted using the Viral RNA Kit (Omega), according to the manufacturer’s instructions. The viral loads were determined by quantitative real-time PCR (qRT-PCR) on a LightCycler 480 II Instrument (Roche) using the SFTSV nucleic acid quantitative detection kit (DaAn Gene Co., Ltd. of Sun Yat-sen University, China), according to the manufacturer’s instructions.

### Detection of infectious SFTSV in the viscera

Frozen viscera were homogenized with zirconia beads in TissueLyser II in 200μl DMEM, centrifuged 15, 000×g at 4°C for 10 min, and 100μl supernatant was taken as the SFTSV stock. The SFTSV stock was diluted 10-fold with DMEM supplemented with 0.5% FBS, and the dilution was inoculated onto a monolayer of Vero cells in 96-well plates. The cells were cultivated for 72 h in a 37°C incubator with 5% CO_2_. The monolayers were fixed with 4% paraformaldehyde (PFA) at room temperature for 30 min. After three 5-min washes with phosphate-buffered saline (PBS), the monolayers were incubated for 1 h with human anti-SFTSV Gn protein mAb. After three 5-min washes with PBS, the monolayers were stained with FITC-conjugated goat anti- human IgG (H-L).

### Immunohistochemistry (IHC)

For fixation of tissues and inactivation of the virus, tissues were submerged for 24 hours in 4% PFA at 4°C. Tissues were paraffin embedded, and 4μm-thick tissues sections were processed for IHC. The tissue sections were incubated with human anti-SFTSV Gn protein mAb. After washing, sections were incubated in biotinylated Rabbit Anti-human IgG (Boster, Wuhan, China), followed by incubation with SABC-Peroxidase solution using a DAB kit (Boster, Wuhan, China). Sections were counter stained with hematoxylin and observed under a microscope. Yellow or brownish-yellow were considered as a positive staining. All sections were scanned by Pannoramic MIDI (The Digital Pathology Company, Budapest, Hungary).

### Statistical analysis

All data were analyzed using IBM SPSS Statistics 24.0 (online). Fetus survival data were analyzed by the *χ*^*2*^ test. The morphological measurements were assessed by the *t* test. For viral loads analysis, the log levels of viral RNA were analyzed by the Mann-Whitney U test.

## Results

### Morphological changes

**Fig 1:**
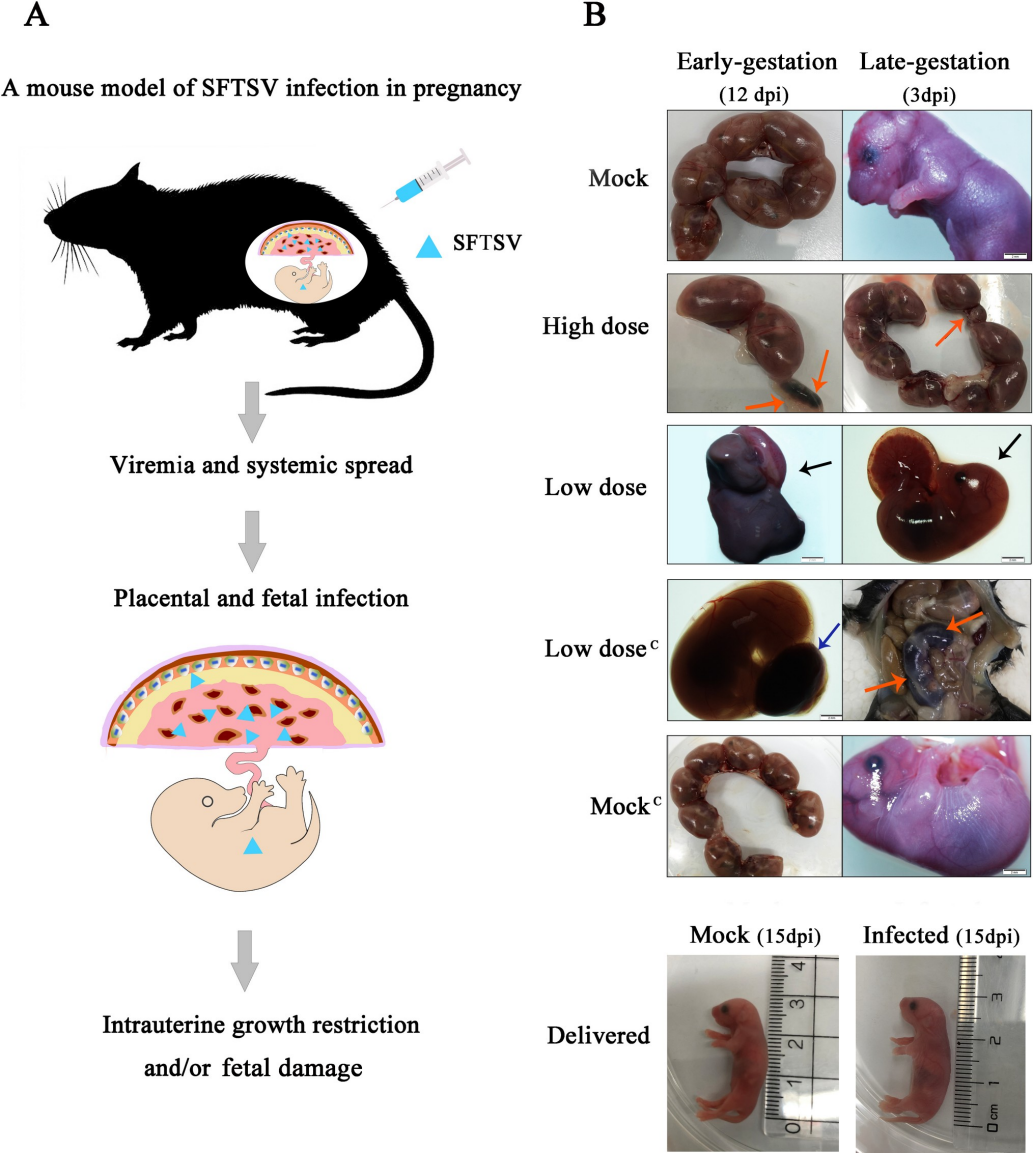
Impact of SFTSV infection on the fetus during pregnancy. **A.** A mouse model of SFTSV infection in pregnancy. **B.** The first and fifth lines are the pregnant dams, which were not infected with SFTSV and the fetuses were normal. The second, third, and fourth lines represent the high dose group, the low dose group, and the low dose group treated with Mitomycin C, respectively. The left column shows infection in early-gestation (E5), the right column shows infection in late-gestation (E14), and the fetuses were dissected and photographed on E17 (12dpi and 3dpi).The red arrow indicates that the fetus had undergone resorption. The blue arrow indicates placental necrosis which appeared as black. The black arrow indicates stillbirths, which were significantly smaller than those of the mock group and shows abnormal skin color. In the bottom two panels are pups that were delivered on day 1 (15dpi). They were from the mock group and the low dose group treated in early-gestation (E5). Scale bars, 2 mm.

**Fig 2:**
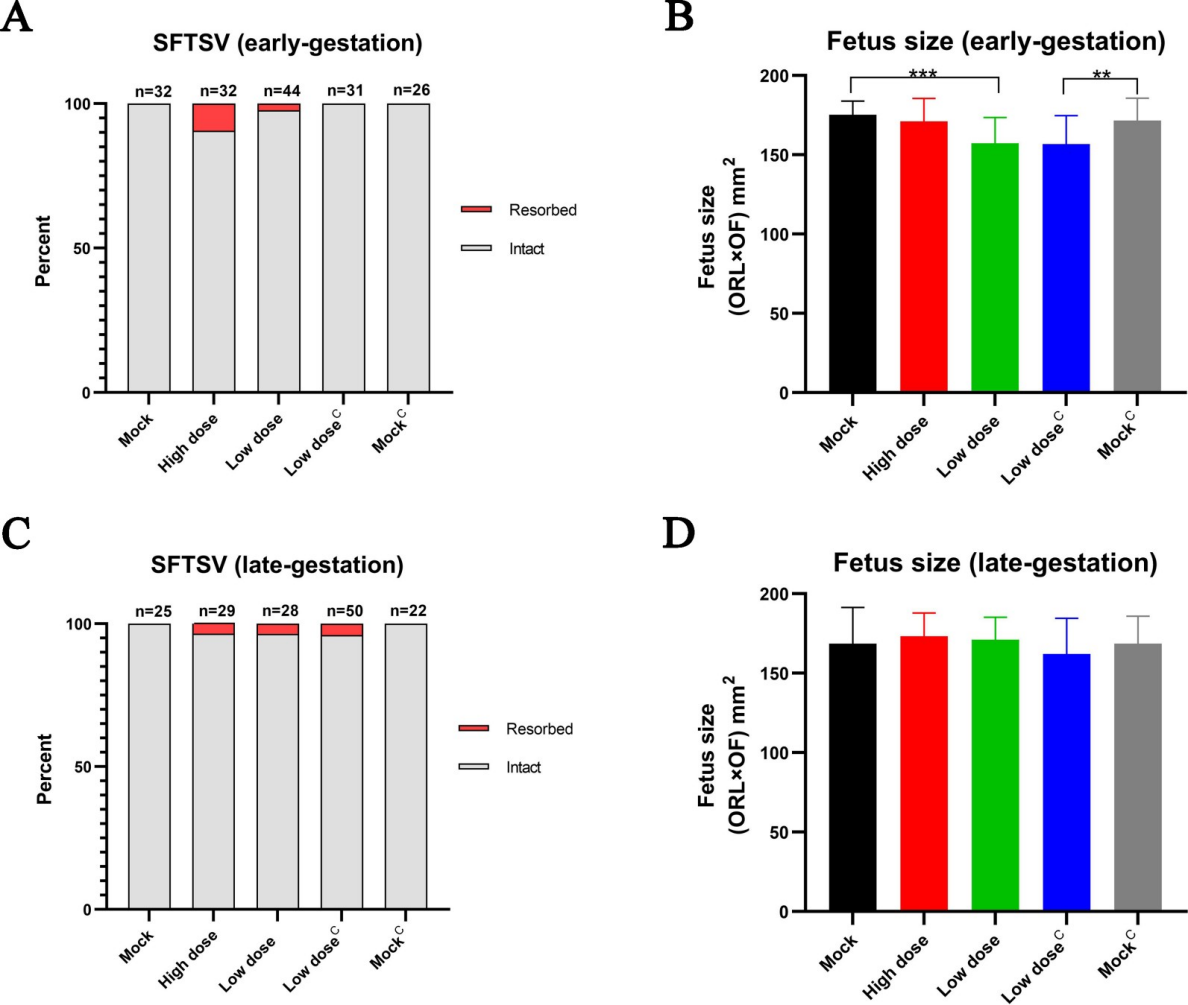
Mortality and size of fetuses after maternal infection with SFTSV. Fetus survival on E17 after infection with SFTSV on E5 (**A**) or E14 (**C**). The n for each group is indicated above each bar. Fetus survival data were analyzed by the *χ*^*2*^ test. Fetus size as assessed by CRL x OF diameter in E17 fetuses following E5 (**B**) or E14 (**D**) infection of pregnant dams with SFTSV. 22 to 48 intact fetuses per group from four or five pregnant dams. The morphological measurements data represent the means ± SD. Fetus size data were analyzed by the *t* test. **p < 0.005; ***p < 0.0005.

In this study, pregnant mice were infected with SFTSV by intramuscular injection (Fig 1A). The SFTSV-infected dams showed no obvious disease symptoms or death. We did not observe an abnormal fetus in the mock group and the mock group treated by mitomycin C, but we did observe that some fetuses had been resorbed, stillborn, and had intrauterine growth restriction (IUGR) in the SFTSV infection groups (Fig 1B). Although some fetuses had been resorbed in SFTSV-infected groups, there was no statistical difference compared with the control group (Fig 2A; 2C). No abnormalities were observed in pups born on E19 in all groups.

The fetus size data showed that infection with a low dose of SFTSV during early-gestation caused fetal IUGR, but this phenomenon was not observed in late-gestation (Fig 2B; 2D). Previous studies suggested that adult C57BL/6 mice infected with SFTSV and treated with mitomycin C had more severe clinical manifestations [20], so this study explored whether mitomycin C can aggravate the degree of fetal damage. The results showed that mitomycin C treatment of pregnant mice had no obvious effect on fetal development (Fig 2B; 2D).

### Detection of viral load

**Fig 3:**
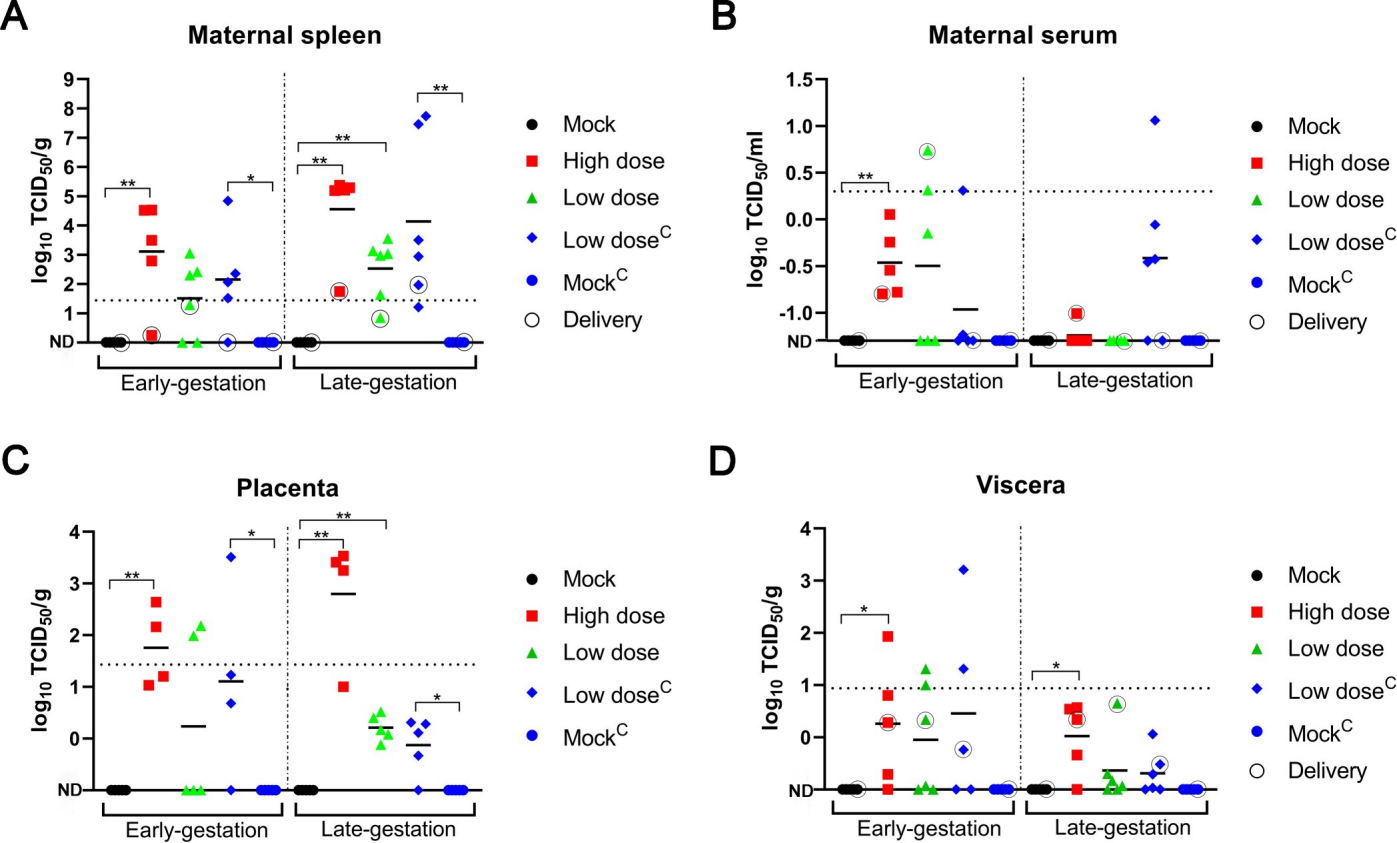
Viral loads in the serum and tissues after maternal infection with SFTSV. Viral loads as measured by qRT-PCR assay from the maternal spleen (**A**), serum (**B**), placenta (**C**) and viscera of fetuses and pups (**D**). Only one placenta and one viscera of a fetus were taken from each dam to detect the viral load. Five groups represented by different colors and shapes during the early-gestation or late-gestation, with black circles representing tissues from dams that had been delivered or pups. Dotted lines of the level represent the limits of detection (LOD) of the qRT-PCR. ND, not detected (below the LOD). The symbol between LOD and ND indicates the presence of SFTSV RNA in the sample but the detection value was not significant due to too few samples. The log levels of viral RNA were analyzed by the Mann-Whitney U test. *p < 0.05; **p < 0.005.

qRT-PCR showed that the spleen of dams infected with SFTSV had abundant SFTSV RNA, especially in late-gestation (Fig 3A). Dams infected with the high dose of SFTSV had higher virus titers in the spleen and placenta than those infected with the low dose. Some of the dams treated with mitomycin C had a higher viral load in the spleen than untreated dams. A low viral load in serum samples was detected in a few dams (Fig 3B).

Of note, the amount of SFTSV RNA within the placenta was greater than in the maternal serum and viscera of fetuses, suggesting that SFTSV preferentially replicates within the placenta (Fig 3B, 3C and 3D). Unfortunately, because the organs of the fetuses and pups were small, the viral load of SFTSV in different organs could not be separately detected. Some dams infected with SFTSV in early-gestation resulted in higher a viral load of fetal viscera than those infected in the late-gestation stage (Fig 3D). Mitomycin C treatment had a significant effect on the viral load in a few placentas and viscera (Fig 3C and 3D). The results showed that SFTSV was vertically transmitted to fetuses and pups through the placental barrier.

### Detection of antibodies to SFTSV

**Fig 4:**
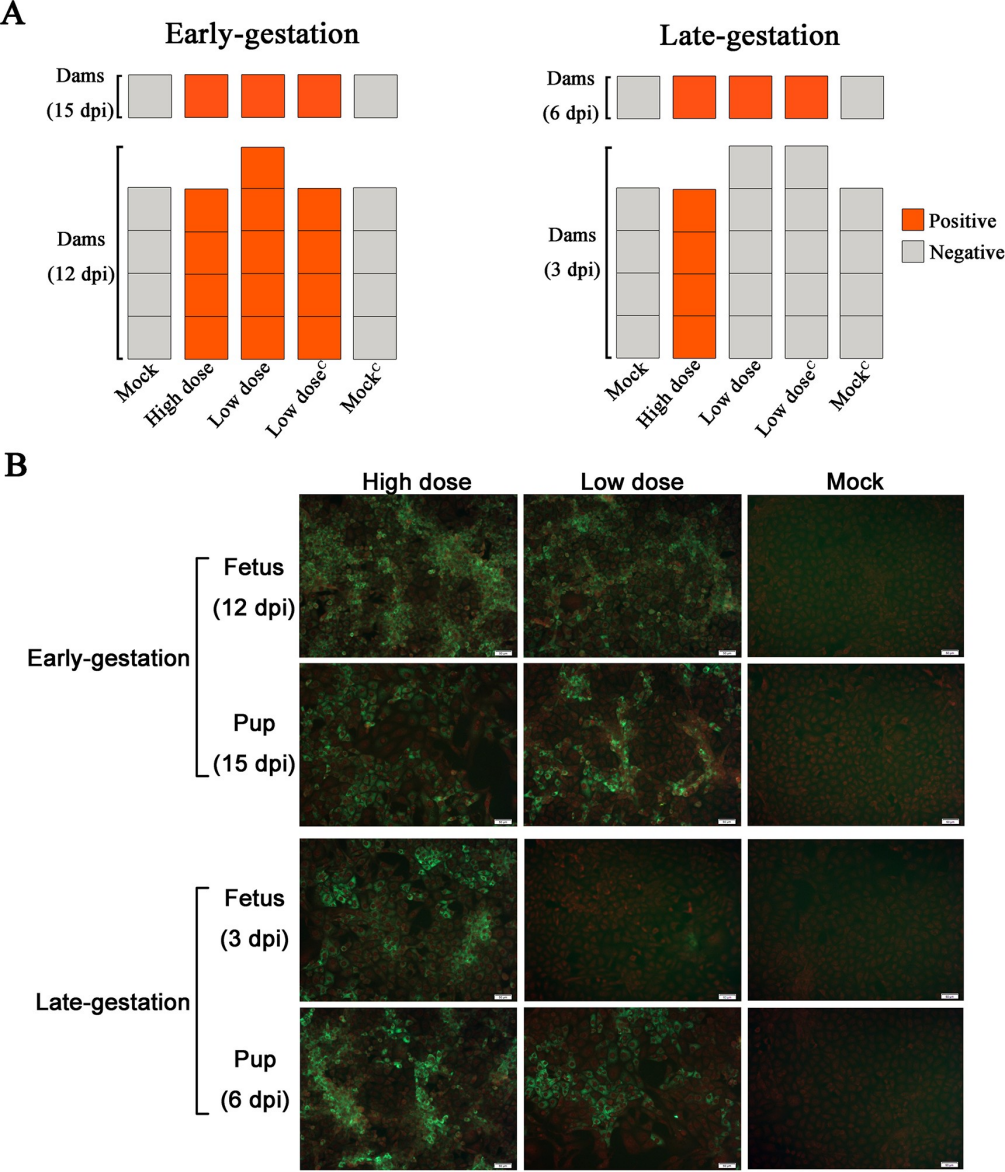
Anti-SFTSV total antibodies in dams and the infectious virus in the viscera of fetuses and pups. **(A)** Anti-SFTSV total antibodies were detected in dams by ELISA. Each panel represents a dam, orange is positive for Anti-SFTSV total antibodies and grey is negative. The upper row of panels represents the dams after normal delivery. (**B)** Detection of infectious SFTSV in the viscera of fetuses and pups. Red indicates the nucleus, and the cytoplasm was labeled with 0.5% Evans blue. Green indicates the distribution of infectious SFTSV in monolayer Vero cells. Images (200×) of cells, scale bars, 50 μm.

SFTSV antibody positive indicated that dams were successfully infected with SFTSV (Fig 4A). SFTSV RNA was detected in the spleens of antibody negative dams infected with SFTSV (Fig 3A). Dams at late-gestation (E14) infected with the high dose (10^6^TCID_50_) SFTSV and sacrificed on E17 (3dpi) were antibody positive. However, dams infected with the low dose (10^3^TCID_50_) SFTSV were antibody negative (Fig 4A). The results suggested that the speed of antibody production in pregnant mice is correlated with the dose of the SFTSV infection.

### Detection of infectious SFTSV in the viscera

The viscera samples with a viral load value greater than ND were used to detect infectious SFTSV using the IFA method. The viscera samples of the mock group served as a control. Infectious SFTSV particles were detected in all viscera samples from SFTSV-infected groups, whereas no infectious SFTSV particles were detected in the viscera samples of the mock group (Fig 4B). The results showed that infectious SFTSV particles can be vertically transmitted to fetuses and pups through the placental barrier.

### Detection of SFTSV antigens in tissues

**Fig 5:**
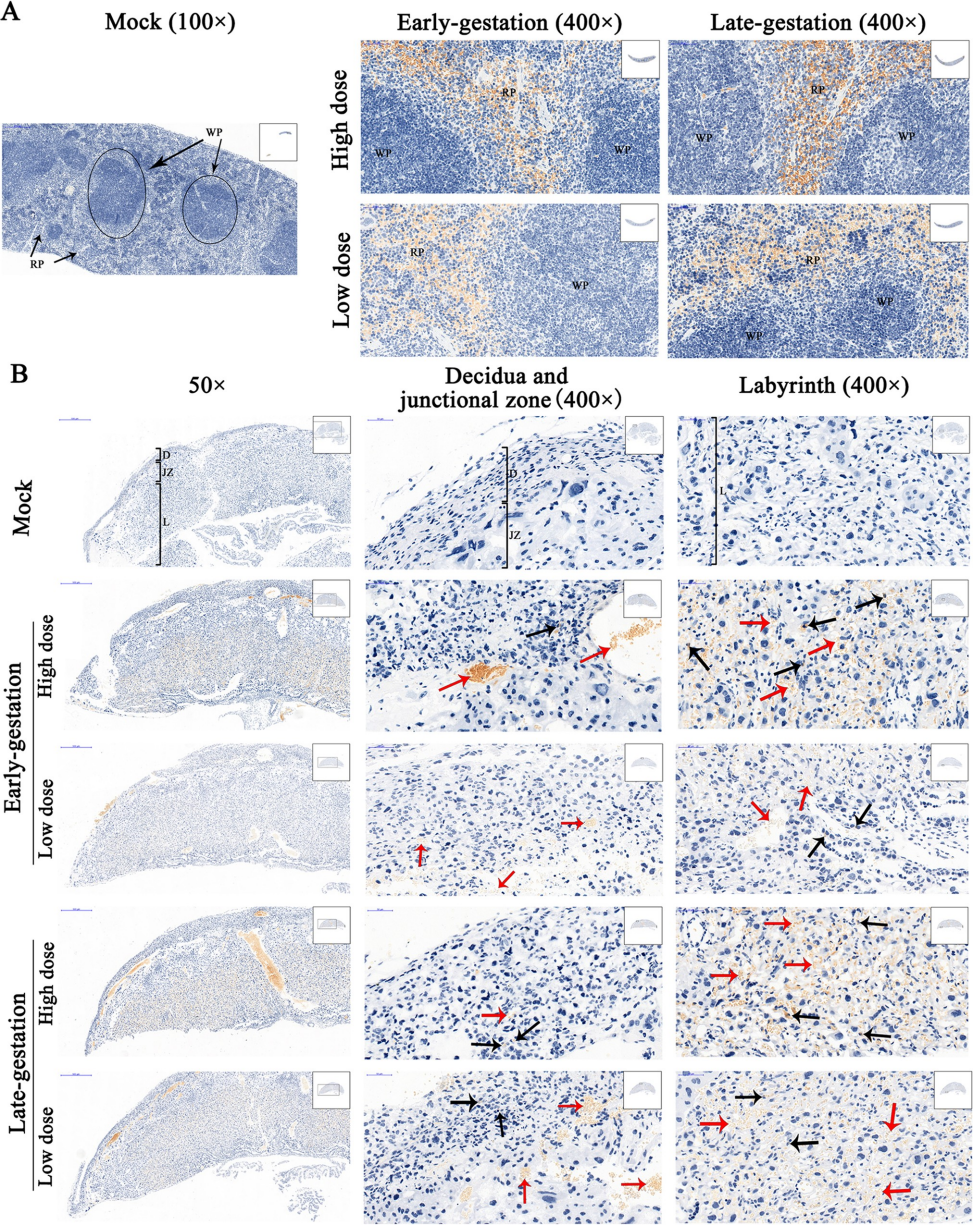
IHC-stained maternal spleen and placenta. Representative IHC-stained tissue sections from SFTSV-infected dams (n = 4 to 5) and mock dams (n = 4) are shown. The pregnant dams were infected with SFTSV or stroke-physiological saline solution (mock) at early-gestation (E5) and late-gestation (E14), and tissues were harvested on E17. Yellow or brownish-yellow are considered as positive staining. (**A**) SFTSV in each group was mainly distributed in the red pulp (RP) of the spleen of dams. The black circle indicates the location of the white pulp (WP). The first column, lower magnification views (100×), scale bars, 200 μm. The other two columns, higher magnification views (400×), scale bars, 50 μm. (**B**) IHC-stained placentas. SFTSV was mainly distributed in the labyrinth (L; third column), and less in the decidua (D) and junctional zone (JZ; middle column). Red arrows indicate red blood cells infected with SFTSV, and black arrows indicate placental cells infected with SFTSV. The first column, lower magnification views (50×), scale bars, 500 μm. The other two columns, higher magnification views (400×) of the decidua, junctional zone, and labyrinth, scale bars, 50 μm.

IHC staining showed that there were abundant SFTSV antigens in the spleen of SFTSV-infected dams, mainly present in the red pulp of the spleen. Viral antigens were almost absent in the white pulp. The results showed that the red pulp in the spleen is the replication site of SFTSV of pregnant mice (Fig 5A). In the mock group, no IHC reaction was detected in the mouse tissues, indicating the specificity of our immunohistochemical method for SFTSV antigens in infected mouse tissues (Fig 5A and 5B).

IHC staining revealed that pregnant mice were infected with SFTSV and there were abundant SFTSV antigens distributed in the placenta. The result of the viral antigen was consistent with the result of qRT-PCR. Viral antigens were observed to be most abundant in the labyrinth, with less distributed in the decidua and junctional zone (Fig 5B). The results suggested that the labyrinth of the placenta of pregnant mice is the replication site of SFTSV.

**Fig 6:**
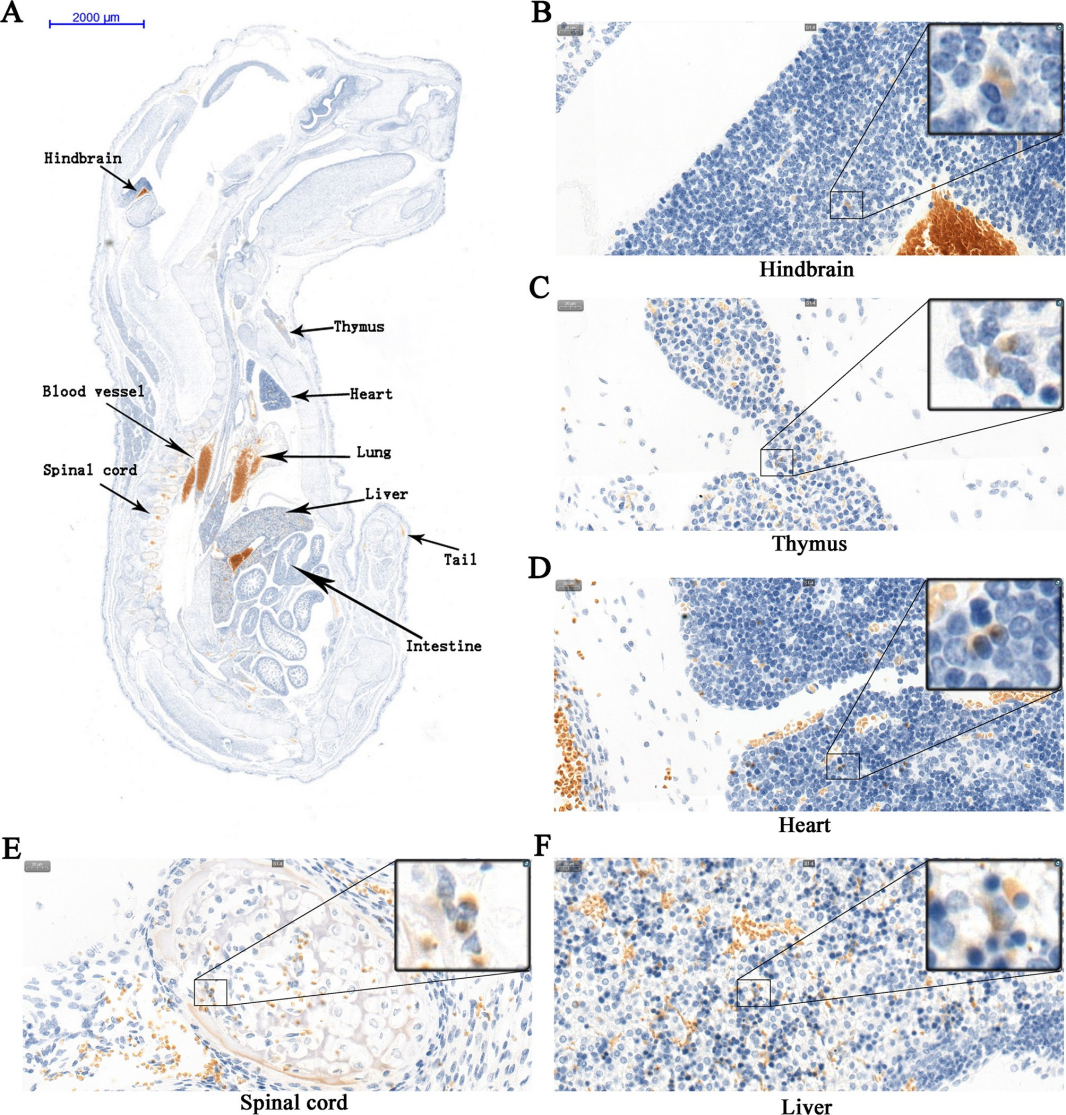
IHC-stained tissues of a fetus infected with SFTSV. The pregnant dams were infected with high dose (10^6^TCID_50_) SFTSV via intramuscular injection at late-gestation (E14), and fetuses were harvested on E17 for IHC. Yellow or brownish-yellow are considered as positive staining. The SFTSV antigen is distributed in the hindbrain (**B**), thymus (**C**), heart (**D**), spinal cord (**E**), liver (**F**), and other organs of the fetus (**A**). (**A**) Images (7×) of fetus, scale bars, 2000 μm. (**B** to **F**) Images (400×) of sections, scale bars, 20 μm.

We found abundant SFTSV antigens in blood vessels, lungs, and liver of congenitally infected fetuses (Fig 6A). In addition, a small amount of SFTSV antigen was observed in multiple organs such as the hindbrain, heart, spinal cord, intestine, tail, and thymus of the fetus (Fig 6). Multiple organs in which the SFTSV antigen was detected in the fetus were SFTSV replication sites. Unfortunately, because the kidney and spleen of the fetuses were small and located on the side, the two organs were missed in the sections, so it was not clear whether the SFTSV antigen existed in these two organs. The results suggested that SFTSV is widely distributed in multiple organs of the fetuses.

## Discussion

Previous studies demonstrated that adult mice of C57BL/6, KM, and BALB/c strains infected with SFTSV did not show visible signs of illness regardless of the inoculation route [21]. Only SFTSV-infected C57BL/6 mice had non-visible features that mimic leukocytopenia, thrombocytopenia, and dysfunction in liver and kidneys that are key clinical presentations of SFTS patients [20]. Although the placenta of humans and livestock are significantly different in morphology from the C57BL/6 mouse placenta, C57BL/6 mice are often used to study placental development, embryogenesis, and abortion during pregnancy [22, 23]. So we used C57 mice as experimental animals. The fully intact placenta forms a physical barrier between the mother and fetus, facilitates nutrient exchange, and protects the developing fetus from microbial threats. The gestational period for mice is 19–20 days, and about E14, the placenta is fully formed [24]. Pregnant mice were inoculated on early-gestation (E5) and late-gestation (E14) to compare the differences in SFTSV infection at different stages of pregnancy. Because humans and livestock are infected with SFTSV mainly through tick bites, we chose intramuscular inoculation for pregnant mice.

In this study, it was observed that some pregnant mice infected with SFTSV showed significant fetal reabsorption and placental damage, whereas this phenomenon was not observed in noninfected pregnant mice. The discrepancy between SFTSV-infected mice and noninfected mice strongly implicates SFTSV infection as a causative agent of fetal reabsorption and placental damage. In early-gestation, the fetuses infected with the low dose of SFTSV showed IUGR, but fetuses infected with high doses of SFTSV did not show this feature. This abnormality may be due to the fact that some fetuses had undergone reabsorption after dams were infected with high dose SFTSV, and only the intact fetuses were measured. In late-gestation, the fetuses did not show IUGR after dams were infected with SFTSV, possibly due to that the fully intact placenta protected the fetus. In addition, we cannot ignore that healthy pregnant mice may also have adverse pregnancy outcomes.

A previous study showed that newborn C57BL/6 mice showed signs of illness and even death after being infected with SFTSV several days later by intracranial (IC) or IP injection [21]. In this study, the pups without signs of illness were likely because the SFTSV carried by the pups were too small and the time during which the pups were observed was too short.

There were differences in the amount of SFTSV RNA in the spleens of dams infected with the same dose of SFTSV at the same time. This was likely due to differences in the level of immunity between individual mice. Although there are differences in the immune system between pregnant and adult mice, SFTSV still persists for a long time after infecting the red pulp of the spleen of the dam.

Like other viruses that cause fetal damage, such as West Nile virus, Zika virus, and RVFV [15, 22, 23], SFTSV had obvious tropism characteristics for the placenta, especially in the labyrinth. The distribution of SFTSV antigens in the placenta was closely related to the structure of the placenta. In mice, the maternal blood passes through trophoblast giant cell (located at the junction of the decidua and the junction zone)–lined channels in the spongiotrophoblast layer (junction zone) to the labyrinth, and the blood collected in the labyrinth is then delivered to the fetus [25]. Therefore, SFTSV reached the placenta with the maternal blood and then largely replicated in the labyrinth.

In early pregnancy, because the placenta was not fully formed, some dams infected with SFTSV resulted in a higher viral load in fetal viscera. In late pregnancy, placentas had a more fully developed placental barrier, and in general exhibited greater resistance to infection. Therefore, there was less SFTSV RNA in the fetal visceral infected by SFTSV in late pregnancy. We noticed that the SFTSV antigen was also present in the brain of the fetuses, which means that the fetuses had a potential risk of developing neurological symptoms.

This study demonstrated that SFTSV can be vertically transmitted to the fetus through the placental barrier of immunocompetent mice, and can result in adverse pregnancy outcomes including placental damage, fetal reabsorption, and IUGR. This finding means that RVFV is not the only phlebovirus that can cause congenital infection of the fetus through the placental barrier [15]. To our knowledge, there are no reports of SFTSV-induced adverse pregnancy outcomes in pregnant women, which may be attributed to the following two reasons. First, SFTS patients were mainly middle-aged and elderly and few young pregnant women were infected with SFTSV. Second, because of the unknown characteristics of SFTSV that could lead to adverse pregnancy outcomes, cases of fetal dysplasia after infection with SFTSV in pregnant women had not attracted the attention of doctors. Therefore, it is necessary to focus on monitoring the pregnancy outcome of women infected with SFTSV. In addition, in order to prevent SFTSV infection, pregnant women should avoid being bitten by ticks and contact with SFTS patients.

## Supporting information

S1 Date Excel spreadsheet containing, in separate sheets, the underlying numerical data and statistical analysis for Figure panels 2A, 2B, 2C, 2D, 3A, 3B, 3C, and 3D. (XLSX)
